# Age-Related Uptake of Heavy Metals in Human Spinal Interneurons

**DOI:** 10.1371/journal.pone.0162260

**Published:** 2016-09-09

**Authors:** Roger Pamphlett, Stephen Kum Jew

**Affiliations:** 1 Discipline of Pathology, Brain and Mind Centre, Sydney Medical School, The University of Sydney, Sydney, New South Wales, Australia; 2 Department of Neuropathology, Royal Prince Alfred Hospital, Sydney, New South Wales, Australia; International Centre for Genetic Engineering and Biotechnology, ITALY

## Abstract

Toxic heavy metals have been implicated in the loss of spinal motoneurons in amyotrophic lateral sclerosis/motor neuron disease (ALS/MND). Motoneuron loss in the spinal anterior horn is severe in ALS/MND at the time of death, making this tissue unsuitable for examination. We therefore examined spinal cords of people without muscle weakness to look for any presence of heavy metals that could make these neurons susceptible to damage. Spinal cord samples from 50 individuals aged 1–95 y who had no clinical or histopathological evidence of spinal motoneuron loss were studied. Seven μm formalin-fixed paraffin-embedded sections were stained for heavy metals with silver nitrate autometallography (AMG^HM^) which detects intracellular mercury, silver or bismuth. Neurons in the spinal cord were classified as interneurons or α-motoneurons based on their site and cell body diameter. Spinal interneurons containing heavy metals were present in 8 of 24 people (33%) aged 61–95 y, but not at younger ages. These AMG^HM^ interneurons were most numerous in the lumbar spinal cord, with moderate numbers in the caudal cervical cord, few in the rostral cervical cord, and almost none in the thoracic cord. All people with AMG^HM^ interneurons had occasional AMG^HM^ staining in α-motoneurons as well. In one man AMG^HM^ staining was present in addition in dorsomedial nucleus and sensory neurons. In conclusion, heavy metals are present in many spinal interneurons, and in a few α-motoneurons, in a large proportion of older people. Damage to inhibitory interneurons from toxic metals in later life could result in excitotoxic injury to motoneurons and may underlie motoneuron injury or loss in conditions such as ALS/MND, multiple sclerosis, sarcopenia and calf fasciculations.

## Introduction

The cause of the motoneuron loss that occurs in the neurodegenerative disorder amyotrophic lateral sclerosis/motor neuron disease (ALS/MND) remains largely unknown [1]. Possibilities include environmental, genetic, or epigenetic factors, or combinations of these, but so far no common cause for the sporadic form of the disease has emerged [2]. Similarly, the genetic or environmental causes of other age-related losses or dysfunctions of motoneurons that occur in normal aging [3,4], the muscle wasting of sarcopenia that occurs in later life [5], or benign fasciculation syndromes [6] are still not identified.

A major difficulty in finding environmental factors that could contribute to motoneuron loss in humans is that in the industrial age an almost limitless number of environmental toxins (toxicants) exist in air, water and soil. This has led to attempts to locate toxicants within the central nervous system in diseases such as ALS/MND, but judging if a patient died with, or from, a toxicant is a challenge. The toxicant could have entered the nervous system years before the disease become clinically apparent and could no longer be detectable at the time of post mortem examination. Furthermore, after death from ALS/MND a severe loss of motoneurons is usually present, and the remaining motoneurons may have survived because they did not contain the toxicant. Finally, a low level of toxicant may be present in only a few neurons within the tissue and so would be missed by chemical analyses [7].

In an attempt to overcome these problems we examined the spinal cords of people who had no evidence of motoneuron damage, to see if any groups of neurons took up toxicants that could cause later motoneuron dysfunction. We used a histochemical technique, autometallography, that visualises some heavy metals within cells [8]. Importantly, this technique detects mercury, a toxicant that has long been implicated in ALS/MND [7]. Unexpectedly, uptake of heavy metals was seen mostly in spinal interneurons, rather than in motoneurons. Inhibitory interneurons damaged by toxicants would lead to a prolonged excitotoxic insult to motoneurons [9], which could result in a range of motoneuron disorders.

## Methods

### Spinal cord samples

Paraffin tissue blocks of spinal cord were available from 50 individuals (24 male, 26 female) with an age range of 1–95 years, who had no clinical or histopathological evidence of spinal motoneuron loss. Individuals included were those where at least one paraffin block from either the cervical or lumbar spinal cord was available. Tissue was obtained from the Department of Forensic Medicine, Sydney, New South Wales, Australia (*N* = 29), and from the Multiple Sclerosis Research Australia Brain Bank (*N* = 21) (Table 1). Causes of death were trauma (*N* = 20), infection (*N* = 7), cancer (*N* = 6), cardiac (*N* = 4), drowning (*N* = 4), infection (*N* = 2), undernutrition (*N* = 2), choking (N = 2), and one each of asphyxia and drug overdose. One individual from the multiple sclerosis brain bank had neuromyelitis optica, and another was found not to have any neurological disorder (see later); of the 19 individuals with neuropathologically-confirmed multiple sclerosis, 17 had secondary progressive, 1 relapsing-remitting, and 1 primary progressive multiple sclerosis. The study was approved by the Human Research Committees of the Sydney Local Health District (Royal Prince Alfred Hospital Zone) and the University of Sydney, and the Office of the New South Wales Coroner. The institutional review board waived the need for written informed consent from relatives of participants since this was a de-identified retrospective study of post mortem tissue.

**Table 1:** Heavy Metal Staining in Spinal Interneurons and Alpha-motoneurons.

Age y	Sex	History	C1-T1			T2-T12			L1-S1		
			No.	INT	αMN	No.	INT	αMN	No.	INT	αMN
95	M	Dementia	12	0	0	0	na	na	0	na	na
89	F	Dementia	16	**++**	0	5	0	0	6	**++**	**+**
85	M	PD	2	0	0	2	0	0	2	0	0
84	F	MS	1	0	0	2	0	0	1	0	0
83	F	MS	3	**++**	**+**	3	0	0	3	**++**	**+**
78	M	Arthritis	1	**++**	0	1	0	**+**	3	**++**	**+**
77	F	PD	0	na	na	2	0	0	6	0	0
74	F	None	3	**+**	0	4	**+**	0	3	**++**	**+**
72	F	MS	1	0	0	1	0	0	1	0	0
72	F	PD	7	**++**	**+**	0	na	na	0	na	na
71	M	MS	2	**+**	0	1	0	0	1	**+**	0
70	M	MS	2	0	0	0	na	na	0	na	na
69	M	None	4	0	0	13	0	0	0	na	na
69	F	MS	1	0	0	3	0	0	0	na	na
68	F	MS	4	0	0	4	0	0	2	**++**	**+**
68	F	MS	1	0	0	3	0	0	0	na	na
67	F	Dementia	1	0	0	0	na	na	0	na	na
66	F	MS	2	0	0	0	na	na	0	na	na
64	M	NMO	1	0	0	4	0	0	2	0	0
64	M	MS	1	0	0	1	0	0	2	0	0
63	M	MS	2	0	0	2	0	0	1	0	0
62	F	MS	1	0	0	2	0	0	1	0	0
62	M	PD	5	0	0	0	na	na	0	na	na
61	F	BD	11	0	0	3	0	0	4	**++**	**+**
61	M	MSA	2	0	0	0	na	na	0	na	na
60	F	MS	2	0	0	1	0	0	1	0	0
59	F	MS	0	na	na	0	na	na	2	0	0
57	F	MS	1	0	0	2	0	0	3	0	0
53	M	None	2	0	0	1	0	0	1	0	0
51	F	MS	3	0	0	0	na	na	2	0	0
50	F	MS	3	0	0	2	0	0	1	0	0
48	M	MS	1	0	0	1	0	0	2	0	0
43	F	Anorexia	6	0	0	6	0	0	6	0	0
40	M	None	3	0	0	0	na	na	4	0	0
40	M	MS	2	0	0	0	na	na	1	0	0
38	F	AN	6	0	0	4	0	0	6	0	0
37	F	None	1	0	0	1	0	0	1	0	0
33	M	None	5	0	0	0	na	na	0	na	na
32	F	None	4	0	0	6	0	0	5	0	0
30	M	None	8	0	0	4	0	0	4	0	0
28	M	Schizophrenia	0	na	na	3	0	0	3	0	0
25	M	None	5	0	0	15	0	0	11	0	0
24	M	None	3	0	0	0	na	na	1	0	0
20	M	Epilepsy	4	0	0	4	0	0	4	0	0
18	F	None	4	0	0	5	0	0	4	0	0
9	M	None	12	0	0	2	0	0	0	na	na
4	M	None	5	0	0	11	0	0	10	0	0
2	M	None	2	0	0	3	0	0	7	0	0
2	F	None	6	0	0	6	0	0	4	0	0
1	F	None	0	na	na	1	0	0	2	0	0

Autometallography staining in the spinal cords of 50 individuals without motoneuron loss, arranged in declining order of age. αMN: α-motoneuron, BD: bipolar disorder, C: cervical cord, INT: interneuron, L: lumbar cord, MS: multiple sclerosis, MSA: multiple system atrophy, na: not applicable; NMO: neuromyelitis optica, No.: number of paraffin blocks, PD: Parkinson’s disease, S: sacral cord, T: thoracic cord, +: 1–2 AMG^HM^ neurons per slide,++: 3 or more AMG^HM^ neurons per slide.

### Histochemical detection of heavy metals

Seven μm sections from each paraffin block were processed routinely and stained with silver nitrate autometallography, which under the protocol used in this study stains the sulphides or selenides of mercury, silver, or bismuth [8]. Briefly, sections were placed in physical developer containing gum arabic, citrate buffer, hydroquinone and silver nitrate at 26°C for 80 min in the dark, then washed in sodium thiosulphate to remove unbound silver. Sections were counterstained with mercury-free hematoxylin and viewed under bright-field microscopy. The silver-coated deposits of these metals in tissues are seen microscopically as black-staining grains, and referred to as autometallography-demonstrable heavy metals (AMG^HM^). In each staining run a positive control section was included of formalin-fixed paraffin-embedded mouse spinal motoneurons that contained mercury following intraperitoneal exposure to mercuric chloride [10].

### Identification of spinal interneurons and motoneurons

Spinal interneurons were identified as neurons less than 24 μm in minimal diameter (measured at the nucleolar level) situated in the grey matter of the anterior horn ventral to the central canal, and α-motoneurons as those >32 μm in minimum diameter in the same region [3,11].

## Results

### Interneurons

**Fig 1:**
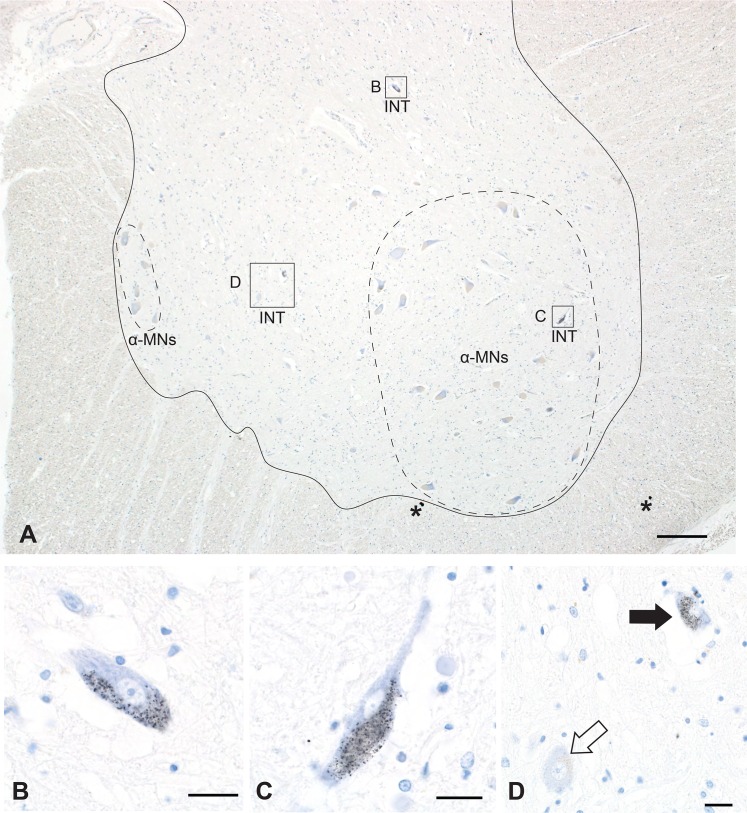
Interneuron Heavy Metal Staining. A. Caudal lumbar spinal cord showing AMG^HM^-stained interneurons (INT) either peripheral to (B, D) or within (C) groups of unstained α-motoneurons (α-MN, within dashed outlines; lateral group right, medial group left) within the grey matter of the anterior horn (the area within the solid outline). Asterisks mark small black dots of artefactual silver staining. Bar = 200 μm. B, C. AMG^HM^ staining (small black grains) is seen in the cytoplasm of two interneurons. Bar = 20 μm. D. Not all interneurons stain with AMG^HM^, as can be seen with this non-stained interneuron (open arrow) adjacent to a stained interneuron (closed arrow). Bar = 20 μm. AMG and hematoxylin.

**Fig 2:**
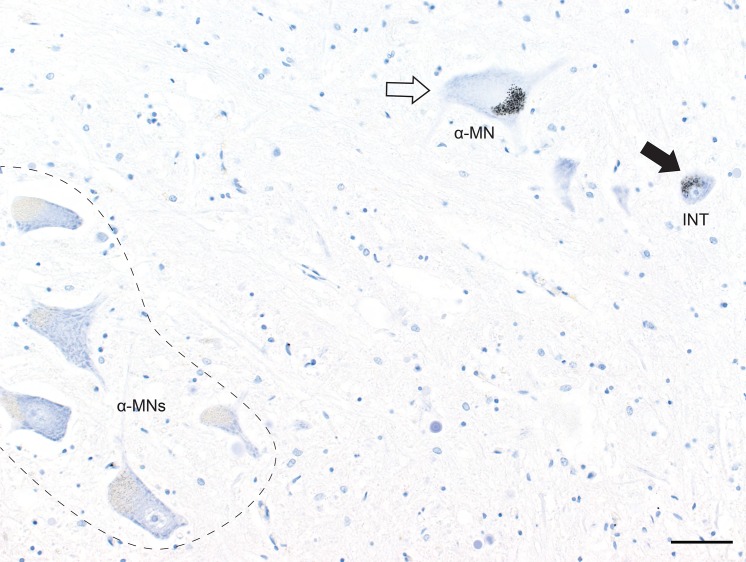
Interneuron and Alpha-motoneuron Heavy Metal Staining. AMG^HM^ staining is seen in the cytoplasm of an α-motoneuron (α-MN) and a nearby interneuron (INT) in the lumbar cord. Most α-motoneurons (within the dashed outline) do not stain for heavy metals. Bar = 50 μm. B.

**Fig 3:**
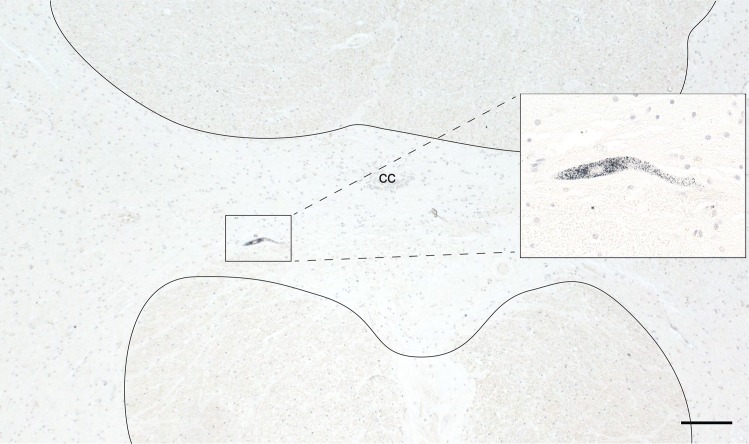
Commissural Neuron Heavy Metal Staining. An occasional AMG^HM^-stained interneuron (within box, magnified in the insert) is seen in the spinal grey matter commissure (the midline region within the solid outlines) in the cervical cord. Bar = 200 μm. AMG and hematoxylin. CC: central canal.

In eight individuals aged between 61 and 95 years (33% of people in this age range) AMG^HM^ staining was present in the cytoplasm of spinal interneurons (Table 1). AMG^HM^ interneurons were situated between, or within, groups of α-motoneurons (Figs 1 and 2). The number of AMG^HM^ interneurons per section varied from 1 to 5. In one individual an AMG^HM^ interneuron was seen in the grey matter commissure adjacent to the central canal (Fig 3). Three individuals with AMG^HM^ interneurons had a history of multiple sclerosis and one each had had dementia, Parkinson’s disease, bipolar disorder, arthritis, and no previous illness.

In some sections AMG^HM^ interneurons were present on only one side of the spinal cord. The number of AMG^HM^ interneurons also varied at different rostro-caudal levels of the spinal cord; often no AMG^HM^ interneurons were seen in a block immediately adjacent to a block containing AMG^HM^ interneurons. Overall, AMG^HM^ interneurons tended to be seen more often in the lumbar compared to the cervical spinal cord. In only one section was an AMG^HM^ interneuron seen in the T2-T12 thoracic spinal cord.

In two individuals where serial blocks had been sampled from C3 to T1, AMG^HM^ interneurons were seen frequently at caudal levels of the cervical cord, as well as in T1, but infrequently at levels rostral to C6. In one individual who had serial blocks sampled from L1-S1 no differences in AMG^HM^ interneuron numbers were apparent between different levels.

### Alpha motoneurons

In individuals where AMG^HM^ interneurons were present, in some sections AMG^HM^ staining was also seen in the cytoplasm of a few spinal α-motoneurons (Fig 2, Table 1). AMG^HM^ stained α-motoneurons were however not present in most sections that contained AMG^HM^ interneurons. In sections where both types of neurons contained heavy metals, numbers of AMG^HM^ interneurons were always greater than numbers of AMG^HM^ stained α-motoneurons.

### Multiple neuronal types and endothelial cells

**Fig 4:**
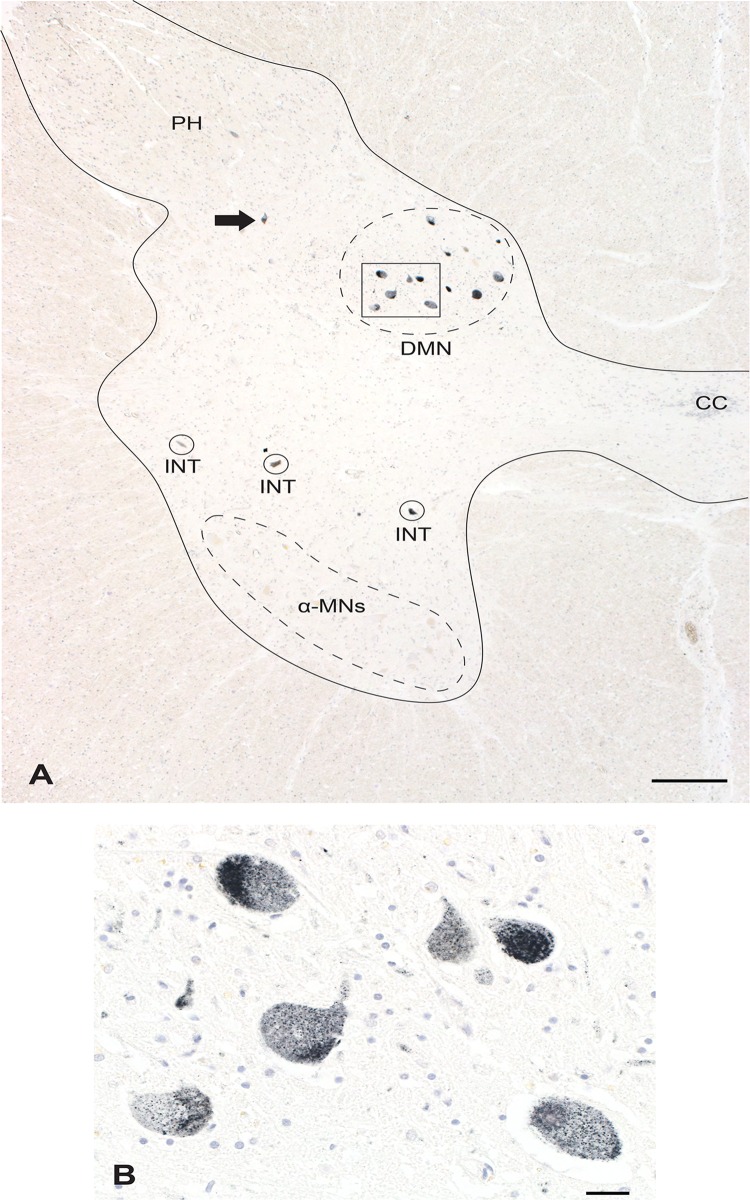
Widespread Heavy Metal Staining. A. In this rostral lumbar spinal cord AMG^HM^ staining is seen in interneurons (INT), in posterior horn sensory neurons (arrow), and in neurons in the dorsomedial nucleus (DMN, Clarke’s column). No α-motoneurons are stained in this section. The grey matter of the cord is within the solid outline. Bar = 500 μm. CC: central canal. B. Higher magnification of the boxed region in A showing heavy AMG^HM^ staining of all dorsomedial nucleus neurons. Bar = 20 μm. AMG and hematoxylin.

**Fig 5:**
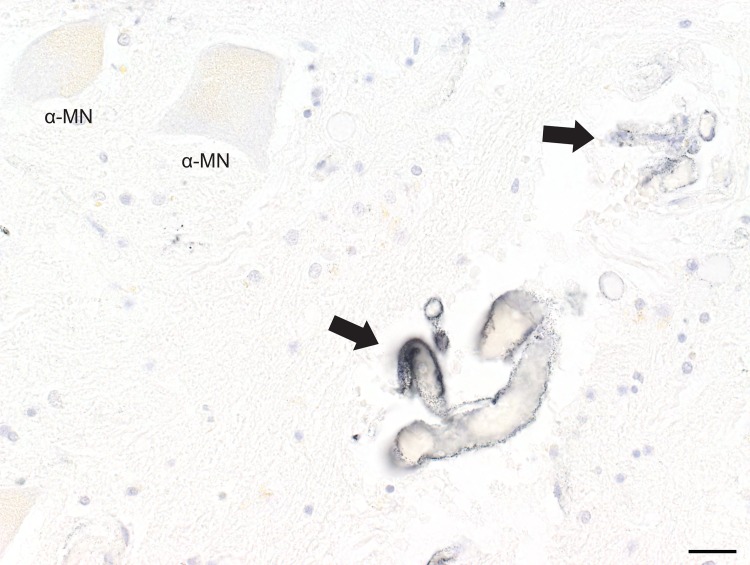
Endothelial cell Heavy Metal Staining. AMG^HM^ staining is seen in the endothelial cells lining two small blood vessels (arrows) in the same spinal lumbar cord as in Fig 4. Two α-motoneurons show no AMG^HM^ staining. Bar = 20 μm. AMG and hematoxylin.

In one 78 year-old man AMG^HM^ staining was present in spinal interneurons and a few α-motoneurons, as well as in a few posterior horn sensory neurons and in all neurons of the dorsomedial nucleus (Clarke’s column) in the caudal thoracic and rostral lumbar cord (Fig 4). AMG^HM^ staining was also present in the endothelial cells of some small spinal cord blood vessels (Fig 5). Early in life there had been a suggestion that he might have multiple sclerosis, but he was later found to have rheumatoid arthritis. Post mortem brain MRI and a neuropathological examination of his brain and spinal cord showed no evidence of CNS pathology.

## Discussion

Heavy metals appear to be commonly taken up by spinal interneurons in advancing age in people who to have no evidence of motoneuron dysfunction. The particular heavy metal present here is likely to be mercury, which is increasingly widespread in air, water and soil due to industrial sources that emit mercury which then enters the mercury cycle [12,13]. Human exposure to the other two heavy metals stained with autometallography, silver and bismuth, is uncommon. Protective mechanisms against heavy metal toxicity, such as binding to metallothionein or selenium in the case of mercury, would protect most individuals against intracellular accumulations of toxic metals. However, those with genetic, epigenetic or environmental susceptibilities to heavy metals [14] could suffer damage to heavy metal-containing interneurons.

Alpha-motoneurons are inhibited by a number of spinal interneurons, either Renshaw cells which inhibit the same α-motoneuron, or 1a interneurons which inhibit α-motoneurons supplying antagonist muscles [15]. Any damage to inhibitory interneurons could result in excitotoxicity to α-motoneurons, a mechanism suggested to underlie motoneuron death in ALS/MND [9]. Neurons containing metal toxins can have decreased physiological function but still survive [10], which could explain why in ALS/MND α-motoneuron numbers may decrease before those of interneurons [16], though others have reported that both sets of neurons degenerate at the same time. The number of interneurons with heavy metals in any one section was small, but interneurons synapse with many α-motoneurons. For example, the ratio of Renshaw cells to α-motoneurons is 1:5 [17], so damage to even a few interneurons could result in widespread α-motoneuron dysfunction.

Heavy metals in interneurons causing excitotoxic damage to α-motoneurons could explain a number of puzzles in ALS/MND. (1) The finding of heavy metals in the interneurons of older individuals fits with the known later age of onset of ALS/MND [18]. (2) The preponderance of interneurons with heavy metals in the lumbar and caudal cervical spinal cord, regions of greatest motoneuron activity during exercise, could be responsible for the ALS/MND postulated risk factor of exercise [19] since active interneurons would be inclined to take up more circulating toxicants. (3) The rarity of a respiratory onset of ALS/MND [20] accords with our finding of the infrequency of heavy-metal containing interneurons in the thoracic spinal cord. (4) Extraocular muscles are rarely affected in ALS/MND [21], and their motor neurons have a different system of inhibitory interneurons which are at some distance from the extraocular nuclei [22]. (5) ALS/MND appears to start at a focal neuroanatomical site and spread from there to adjacent motoneurons [23]. The number of initiation sites could determine the rate of diseases progression [2], and having more interneurons containing heavy metals could underlie increased rates of progression.

Heavy metal staining was widespread in the spinal cord of one individual, involving interneurons, motor neurons, sensory neurons, and neurons in the dorsomedial nucleus (Clarke’s column). Heavy metal staining in endothelial cells suggests ongoing exposure to the metal, though the individual had no clinical features of heavy metal toxicity. Clarke’s column neurons have long been known to be involved in ALS/MND [24], and pathological TDP43 inclusions are seen in this nucleus in one-third of patients [25]. Sensory abnormalities can often be found on nerve conduction studies in ALS/MND, consistent with our finding of heavy metals in some posterior horn neurons [26]. It seems possible therefore that toxicants could determine which types of neurons are affected in ALS/MND and could therefore underlie some of the variability in neuroanatomical phenotype that is typical for the disease [27].

Of interest was the finding of an occasional α-motoneuron with cytoplasmic heavy metal staining, often with a nearby interneuron showing similar staining. The dual stress of excitotoxicity and toxicant load could well be too much for the α-motoneurons to bear, and cell damage and death could result, especially if some susceptibility to metal toxicity were present. This could initiate the spread of disease, particularly since the metal in question is likely to be mercury. Mercury has many of the toxic properties which are suspected to underlie ALS/MND [28]. These include an ability to activate retroelements [29], some of which, such as human endogenous retrovirus-K, have been implicated in the spread of ALS/MND [30].

Spinal motor neuron numbers decline with age [3], and this loss is most readily apparent after the age of 60 years, when motoneuron counts can be up to 50% lower than in early adult life [4]. Small neurons in the dorsomedial portion of the spinal anterior horn are most severely lost in aging, more so than large α-motoneurons [31]. These small neurons are in the same region of the spinal cord as many of the heavy metal-staining interneurons in our study. Age-related losses of interneurons and of α-motoneurons may underlie the muscle wasting of sarcopenia, since muscle denervation is a prominent feature of this condition [5]. Of note, spinal reciprocal inhibition decreases with age [32], as might be expected with damaged interneurons. Furthermore, in sarcopenia the decrease in muscle mass is greater in the lower than the upper limbs, which is counterintuitive since the lower limbs are usually exercised more [33]. There was a tendency in our study to see more heavy metal-containing spinal interneurons in the lumbar than the cervical spinal cord, which could account for this disproportionate decrease in lower limb muscle mass in sarcopenia.

Fasciculations, spontaneous intermittent contractions of a portion of a muscle, are a clinical hallmark of ALS/MND, but can be seen as a benign condition in later age when restricted to muscles below the knee [6]. Fasciculations are thought to be generated by abnormal activity of α-motoneurons, possibly from axonal damage, though the temporary increase in fasciculations produced by intense exercise suggests a more central mechanism may be in operation [6]. Such a mechanism may be toxicant-induced reduced interneuron inhibition of α-motoneurons, since our data show heavy metal uptake into interneurons is age-related and that interneurons in the lumbar spinal cord are particularly affected.

Spinal α-motoneuron and interneuron loss is not uncommon in multiple sclerosis, even in regions distant from plaques [34,35]. People with multiple sclerosis can have focal asymmetric wasting of muscles, especially in the hand [36]. Occasionally, multiple sclerosis and ALS/MND co-exist, raising the possibility of a shared pathogenesis for motoneuron loss in these conditions [37]. Our finding of heavy metals in spinal interneurons raises the possibility that toxicant uptake in these interneurons may be the factor underlying motoneuron loss in both these conditions, though α-motoneuron loss in multiple sclerosis does not appear to be age-related [36].

**Fig 6:**
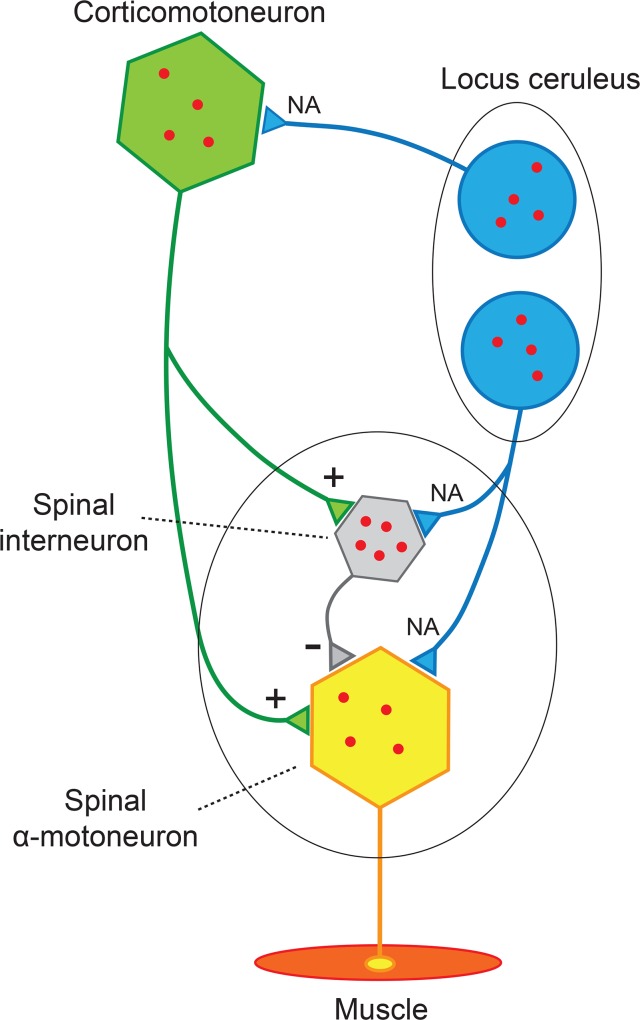
Neurons of the Motor System Predisposed to Heavy Metal Uptake. Human spinal interneurons, α-motoneurons, corticomotoneurons, and locus ceruleus neurons are prone to take up circulating heavy metals. Differences in toxicant uptake between these neurons could explain some of the phenotypic variability of ALS/MND, and underlie a spectrum of conditions that include motoneuron loss in normal aging, sarcopenia, benign fasciculation syndrome, and amyotrophy in multiple sclerosis.

Spinal interneurons can now be added to three other types of neurons (α-motoneurons, corticomotoneurons, and locus ceruleus neurons) which human studies using autometallography have indicated are prone to take up heavy metals [38] (Fig 6). These neurons are part of (or in the case of locus ceruleus neurons, have a major influence on [39]) spinal and cortical motoneurons. We propose that exposures to single or multiple toxicants with uptake of these into different neurons, combined with susceptibilities to the toxicants, could underlie a spectrum of disorders of the motor system. Differences in susceptibility to mercury toxicity, for example, could arise due to genetic polymorphisms affecting the mercury-binding protein metallothionein [40], epigenetic differences [14], or low selenium levels [41], any of which could stimulate mercury neurotoxicity.

In conclusion, heavy metals appear to accumulate during aging in human spinal interneurons, as well as in a few α-motoneurons. Toxicant-induced dysfunction of spinal interneurons could lead, especially in susceptible individuals, to damage or loss of α-motoneurons. This could manifest in a spectrum of motoneuron disorders which include ALS/MND, sarcopenia, benign fasciculation syndrome, and amyotrophy in multiple sclerosis.
